# Functional consequences of enhanced expression of STIM1 and Orai1 in Huh-7 hepatocellular carcinoma tumor-initiating cells

**DOI:** 10.1186/s12885-019-5947-z

**Published:** 2019-07-31

**Authors:** B. Karacicek, Y. Erac, M. Tosun

**Affiliations:** 1 Izmir Biomedicine and Genome Center (IBG), Dokuz Eylul University, 35340 Izmir, Turkey; 2 Department of Pharmacology, Faculty of Pharmacy, Ege University, 35100 Izmir, Turkey; 3 Department of Pharmacology, School of Medicine, Izmir University of Economics, 35330 Izmir, Turkey

**Keywords:** HCC, SOCE, TIC, STIM1, Orai1, Ca^2+^

## Abstract

**Background:**

The endoplasmic reticulum (ER) Ca^2+^ sensor, stromal interaction molecule1 (STIM1) activates the plasma membrane (PM) channel Orai1 in order to mediate store-operated Ca^2+^ entry (SOCE) in response to ER store depletion. Enhanced expression of STIM1 in cancer tissue has been associated with poor patient prognosis. Therefore, this study investigated the functional consequences of enhanced expression of STIM1 and Orai1 in a tumor-initiating subpopulation of Huh-7 hepatocellular carcinoma (HCC) cells that express epithelial cell adhesion molecule (EpCAM) and Prominin 1 (CD133).

**Methods:**

We performed qRT-PCR, intracellular Ca^2+^ monitoring, protein analyses, and real-time cell proliferation assays on EpCAM(+)CD133(+) subpopulation of tumor-initiating Huh-7 HCC cells expressing high levels of STIM1 and/or Orai1. Statistical significance between the means of two groups was evaluated using unpaired Student’s *t-*test.

**Results:**

Enhanced STIM1 expression significantly increased ER Ca^2+^ release and proliferation rate of EpCAM(+)CD133(+) cells.

**Conclusion:**

STIM1 overexpression may facilitate cancer cell survival by increasing ER Ca^2+^-buffering capacity, which makes more Ca^2+^ available for the cytosolic events, on the other hand, possibly preventing Ca^2+^-dependent enzymatic activity in mitochondria whose Ca^2+^ uniporter requires much higher cytosolic Ca^2+^ levels.

## Background

Hepatocellular carcinoma (HCC) appears to be the third leading cause of cancer-related deaths worldwide [1–11]. The primary issue in HCC cases is the high recurrence rates [12] possibly due to the existence of chemotherapy-resistant tumor-initiating cell (TIC) subpopulations [13]. Tumor-initiating cells constitute 0.01–1% of tumor mass [14, 15]. These cells express certain cell surface antigens used for separating them from other cell types within the heterogeneous tumor cell lines [16]. Epithelial cell adhesion molecule (EpCAM) and Prominin 1 (CD133) are frequently used to identify Huh-7 human HCC TICs [17, 18] as NOD/SCID mice developed tumor after receiving Huh-7 cells expressing these two antigens [19].

SOCE, a major Ca^2+^ influx through Ca^2+^-release activated Ca^2+^ (CRAC) channels in non-excitable cells [20–25], has been shown to be operational both in normal hepatocytes and HCC [26]. SOCE components are the ER-resident Ca^2+^ sensor stromal interaction molecule 1 (STIM1) [27] and the PM Ca^2+^ channel Orai1 [28–30]. The roles of STIM1 and Orai1 in carcinogenesis, tumor initiation, proliferation and metastasis have recently attracted significant attention [27, 31]. Indeed, altered expression of STIM1 and Orai1 is a hallmark of many cancer types, suggesting their potential value as prognostic biomarkers in cancer [27, 32–35].

TICs appear to be responsible for high recurrence rates as well as for chemoresistance [36]. HCC cells are a non-excitable cell type, where SOCE plays a crucial role in Ca^2+^ homeostasis and signaling [37]. In many cancer types including HCC, enhanced expression of STIM1 and Orai1 have been shown to enhance carcinogenesis including proliferation, migration and invasion processes [26, 38, 39]. Previous studies have reported that STIM1 and Orai1 molecules mix at a specific ratio to encode functional CRAC channel assembly [40, 41]. Based on crystallographic and electrophysiological studies, STIM1 exists as a dimer under resting conditions, and binds to Orai1 in a nonlinear fashion such that all six Orai1 binding sites must be occupied for the activation of SOCE [42]. However, the structural basis of STIM1 interaction with Orai1 within the channel assembly is not known. Therefore, the purpose of this study is to investigate the functional impact of altered stoichiometry of STIM1 and/or Orai1 by employing overexpression plasmid vectors on intracellular Ca^2+^ dynamics as well as carcinogenic properties of Huh-7 EpCAM(+)CD133(+) cells.

## Methodsn

### Cell culture

Human HCC cell lines (Huh-7) were provided by Dr. Ozturk (IBG İzmir), originally from Dr. Jack Wands Laboratory (Massachusetts General Hospital, Boston, MA) as a gift, and tested for authenticity via DNA profiling (Applied Biosystem’s Identifier kit, PN 4322288) at DNA Sequencing & Analysis Shared Resource, University of Colorado Cancer Center. The authenticity was reconfirmed by Idexx Bioresearch Company (Germany) just before initiating our studies. In addition to these, the cells have been also checked regularly in our laboratory for mycoplasma contamination by using MycoAlert Mycoplasma Detection kit (Lonza). Parental Huh-7 HCC cells and the sorted cells after Fluorescence Activated Cell Sorting (FACS, FACSAria III, BD) were maintained in complete growth medium (Dulbecco’s modified Eagle medium, DMEM, Sigma) containing 10% heat-inactivated fetal bovine serum (FBS, Biowest), 2 mM L-glutamine (Sigma) and 0.1 mM non-essential amino acids (Sigma).

### Selection of EpCAM(+)CD133(+) and EpCAM(−)CD133(−) Huh-7 cells with FACS

Huh-7 HCC cells were trypsinized, washed, and resuspended in FACS buffer (1XPBS, 1 mM EDTA, 25 mM HEPES, 1% FBS) and filtered through 0.2 μm filter. Cells were passed through cell strainers with pore diameters of 100 and 30 μm (Miltenyi) to eliminate cell aggregates. Cells (15 × 10^6^) were centrifuged to obtain pellets, then, resuspended in 105 μl FACS buffer followed by reincubation with 30 μl FcR blocking reagent (Miltenyi), 15 μl EpCAM-FITC (Miltenyi) and 15 μl CD133-PE (Miltenyi) for 10 min on ice. After incubation, cells were washed with FACS buffer and sorted via a fluorescence-activated cell sorter (FACS Aria III, BD Biosciences). Cells with and without EpCAM and/or CD133 were separately collected inside FBS containing tubes. After sorting, purity percentages for EpCAM(+)CD133(+) were determined with FACSCalibur (BD Biosciences) on the fifth day.

### Transfection of EpCAM(+)CD133(+) Huh-7 cells with STIM1 and Orai1 overexpression plasmids

Cells were seeded on 6 well-plate (10^5^ cells/well) and transfection was performed after 24 h with X-tremeGENE HP DNA Transfection Reagent (Roche). Following removal of the cell media, serum-reduced media (Opti-MEM) were added and incubated for additional 1 h. 100 μl Opti-MEM, 1.5 μg plasmid DNA (MO70-STIM1-eYFP, pDEST501-Orai1-CFP and pCMV6 empty vector as a control) and 1 μl X-tremeGENE HP DNA Reagent-containing transfection mix was added to each well and incubated for 30 min at room temperature. Transfection mix was added on the cells dropwise and shaked gently. Plasmids were gently provided by Dr. M Trebak (Penn State University).

### RNA isolation and cDNA synthesis

Cells were seeded on 6-well plate (15 × 10^4^/well). Total RNA was isolated by using High Pure RNA Isolation (Roche) according to the manufacturer’s instructions. cDNA synthesis from the total RNA samples were performed by using Transcriptor First Strand cDNA Synthesis Kit (Roche) according to the manufacturer’s instructions.

### Real-time quantitative RT-PCR (qRT-PCR)

FastStart DNA Master SYBR Green I kit was used in real-time qRT-PCR experiments performed (LightCycler 1.5, Roche Applied Science). Primer sequences are shown in Table 1. All expression levels were normalized to that of internal *18S rRNA* ([*Target gene*]/[18S rRNA] × 100).

**Table 1:** Oligonucleotide sequences of qRT-PCR primers

Target Accession number	Gene	Sequence (5′-3′)	Amplicon size (bp)
NM_001277961	*STIM1*	F: AGC AGA GTT TTG CCG AAT TG	132
		R: ATC ACT TTC TTC CAC ATC CAC AT	
NM_032790.3	*Orai1*	F: CAG AGT TAC TCC GAG GTG ATG AG	119
		R: GAG AGC AGA GCC GAG GTC C	
NR_003286	*18S rRNA*	F: CGA CGA CCC ATT CGA ACG TCT	312
		R: GCT ATT GGA GCT GGA ATT ACC G	
NM_000927.4	*MDR1*	F: CAG AGG GGA TGG TCA GTG TT	197
		R: TCA TAG GCA TTG GCT TCC TT	

*F* forward, *R* reverse, *bp* base pair

### Protein isolation and Western blot

Protein isolation was performed on 15 × 10^4^ cells seeded on 6-well plate by cOmplete Lysis-M, EDTA-free (Roche) according to the manufacturer’s instructions. Protein extracts, separated by SDS-PAGE were transferred onto PVDF membranes, then, incubated with antibodies targeted against STIM1 (3 μg/μl, Abcam), Orai1 (1:750, Abcam) and β-actin (1:5000, Sigma) overnight at 4 °C. Membranes were incubated with secondary antibodies (1:5000, anti-rabbit or anti-mouse, LI-COR) for 1 h via shaking at room temperature. Protein bands were visualized in an infrared imager (Odyssey, LI-COR) based on the appropriate channel properties (680RD or 800CW) of secondary antibodies.

### Intracellular Ca^2+^

Cells seeded on circular coverslips were loaded with 5 μM Fura-2/AM (Molecular Probes) in HEPES-buffered saline. Changes in intracellular Ca^2+^ levels were monitored via a front-surface spectrofluorometer (PTI QM8/2005) as described earlier [43].

### Real-time monitoring of proliferation by real-time cell analyzer (RTCA)

Real-time label-free impedance-based monitoring of cellular proliferation assay was performed by using xCELLigence MP (Roche Applied Science). Transfected cells were incubated in 6-well plates for 48 h. After the incubation period, 5000 cells/well were seeded in E-plate 96. Cell proliferation was monitored at every 15 min for 72 h. Changes in proliferation rate were expressed as “cell index” (RTCA software 1.2.1, Roche Applied Science).

### Data analysis

Data expressed as mean ± standard error of the mean (S.E.M.). *“n”* denotes the number of samples. Statistical significance between the means of two groups was evaluated using Student’s *t-*test (unpaired data). Significance was accepted at 0.05 level of probability.

## Results

### Selection of EpCAM(+)CD133(+) and EpCAM(−)CD133(−) Huh7 cells

**Fig. 1:**
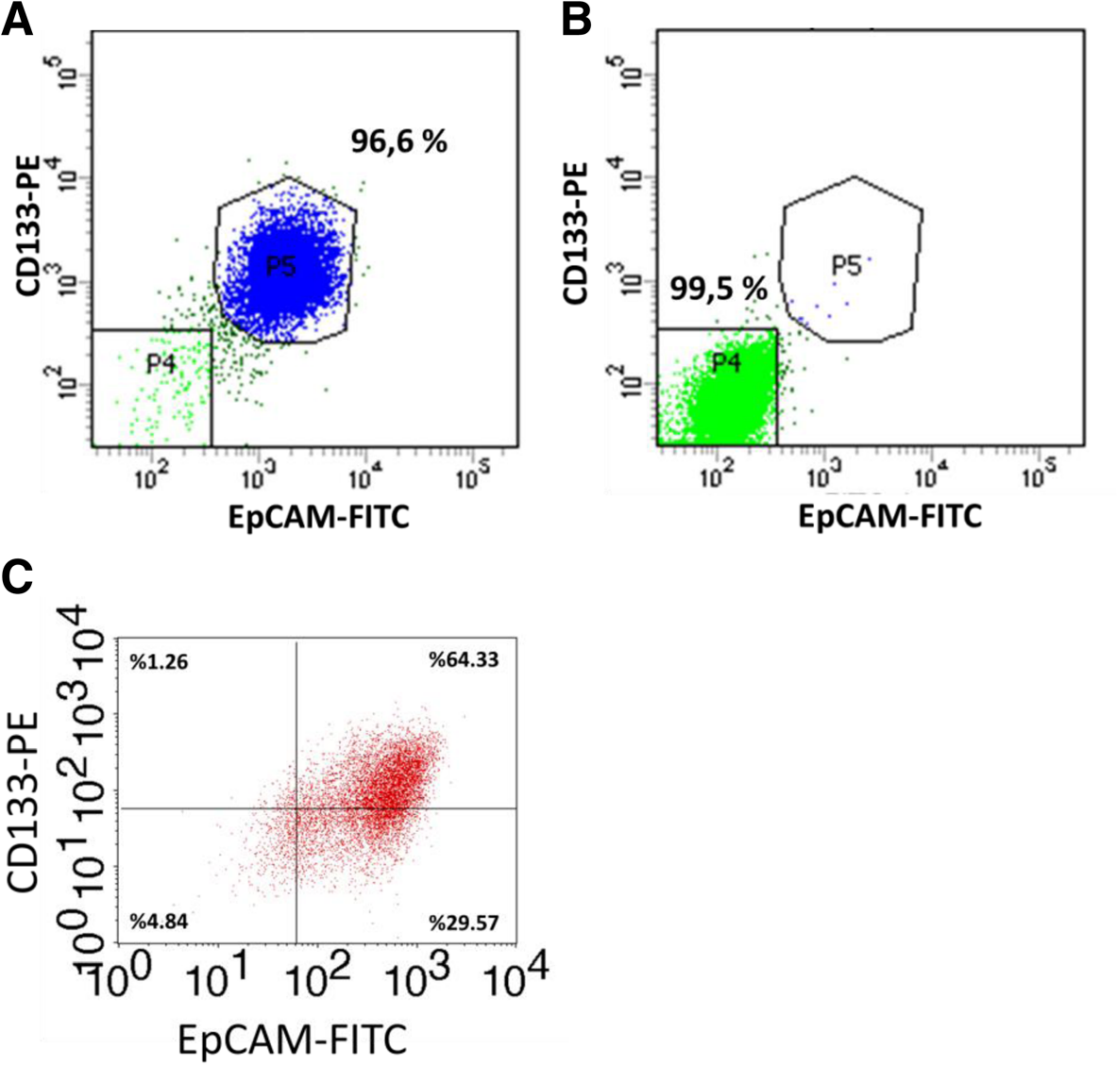
EpCAM and CD133 antigen-expressing Huh-7 cell distribution after separation. **a** EpCAM(+)CD133(+) 96.6% in Day 0, P5 gate for EpCAM(+)CD133(+), (**b**) EpCAM(−)CD133(−) Huh-7 cells 99.5% in Day 0, P4 gate for EpCAM(−)CD133(−) and (**c**) EpCAM(+)CD133(+) in Day 5. EpCAM-FITC: fluorescein isothiocyanate conjugated EpCAM, CD133-PE: Phycoerythrin conjugated CD133

EpCAM(+)CD133(+) and EpCAM(−)CD133(−) Huh-7 cells were selected from a parental Huh-7 cell line via a FACS. Figure 1a and b show the percentages of EpCAM(+)CD133(+) and EpCAM(−)CD133(−) cells after sorting (Day 0) and on the 5th day (Day 5) Fig. 1c. On Day 5, as cells reach about 70% confluency in order to be ready for the transfection procedure, the EpCAM(+)CD133(+) cell population decreased from 96.6 to 64.3%.

**Fig. 2:**
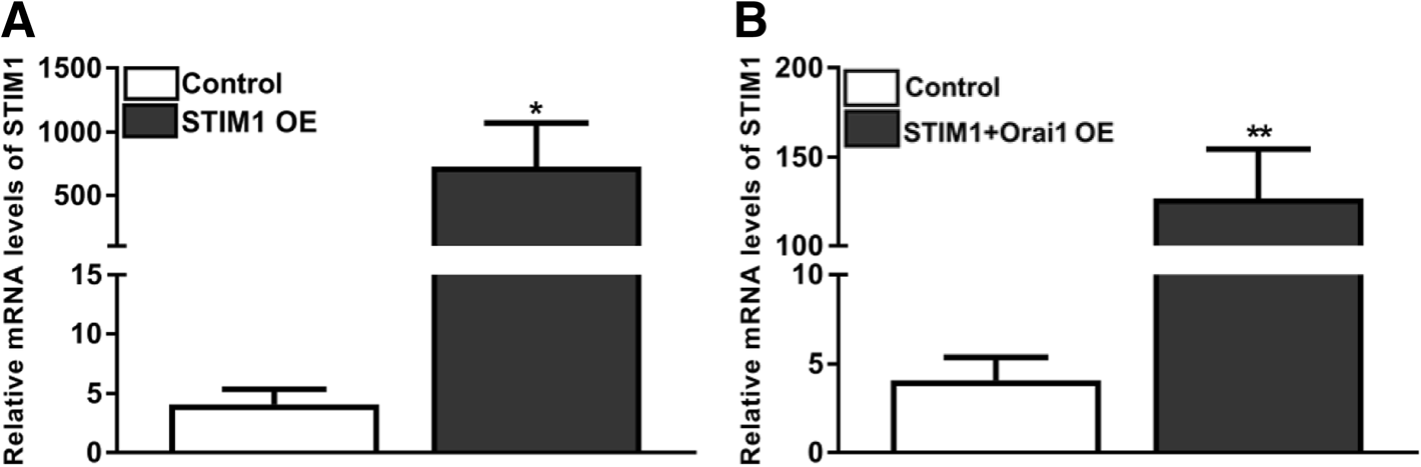
STIM1 mRNA expression levels in control and plasmid-transfected EpCAM(+)CD133(+) cells. Shown are (**a**) control vs. STIM1-OE and (**b**) control vs. STIM1 + Orai1-OE (Target gene/*18S rRNA*x10^2^; *p < 0.05; **p < 0.01, Student *t*-test, unpaired data, *n* = 4)

In addition to microscopic examinations, overexpression (OE) efficiency of STIM1 and Orai1 in all experimental conditions (STIM1-OE, Orai1-OE, STIM1 + Orai1-OE) on EpCAM(+)CD133(+) cells was confirmed via real time qRT-PCR. STIM1 and Orai1 expression levels were not significantly different between EpCAM(+)CD133(+) and EpCAM(−)CD133(−) cells (data not shown). In STIM1-OE and STIM1 + Orai1-OE EpCAM(+)CD133(+) cells (Fig. 2) STIM1 increased both in STIM1-OE (*p* < 0.05, Fig. 2a) and STIM1 + Orai1-OE cells (**p < 0.01, Fig. 2b) as expected.

**Fig. 3:**
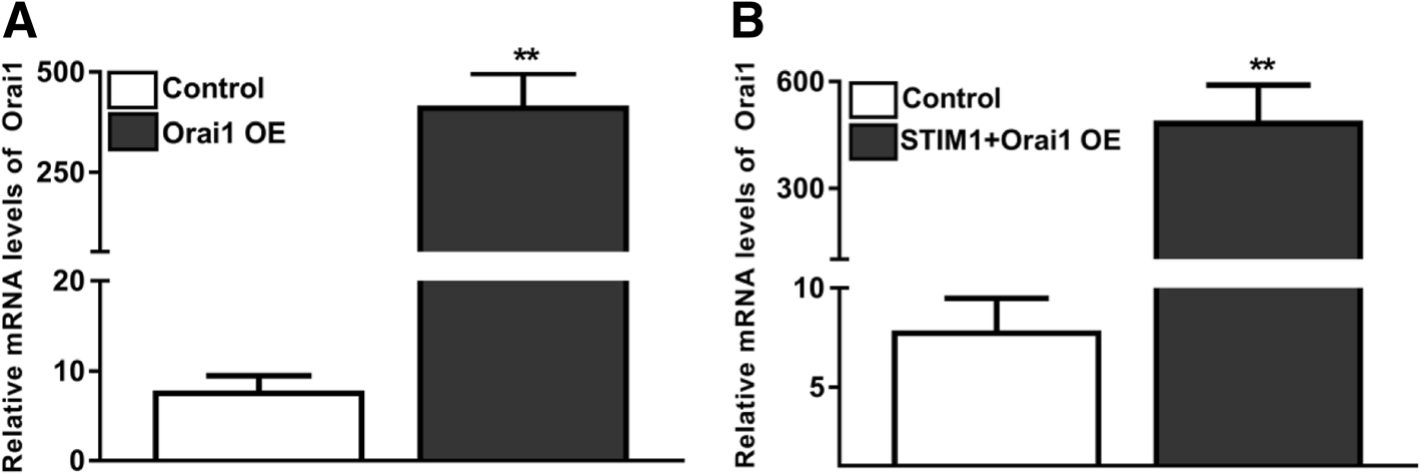
Orai1 mRNA expression levels in plasmid-transfected EpCAM(+)CD133(+) cells. Shown are (**a**) control vs. Orai1-OE and (**b**) control vs. STIM1 + Orai1-OE (Target gene/*18S rRNA*x10^2^; **p < 0.01, Student *t*-test, unpaired data, n = 4)

Orai1 mRNA level increased in Orai1-OE (**p < 0.01, Student *t*-test, unpaired data *n* = 4, Fig. 3a) and STIM1 + Orai1-OE (**p < 0.01, Student *t*-test, unpaired data, n = 4, Fig. 3b) EpCAM(+)CD133(+) cells (Fig. 3) comparable to the control, which is similar to that of STIM1 mRNA expression levels revealed in previous data.

**Fig. 4:**
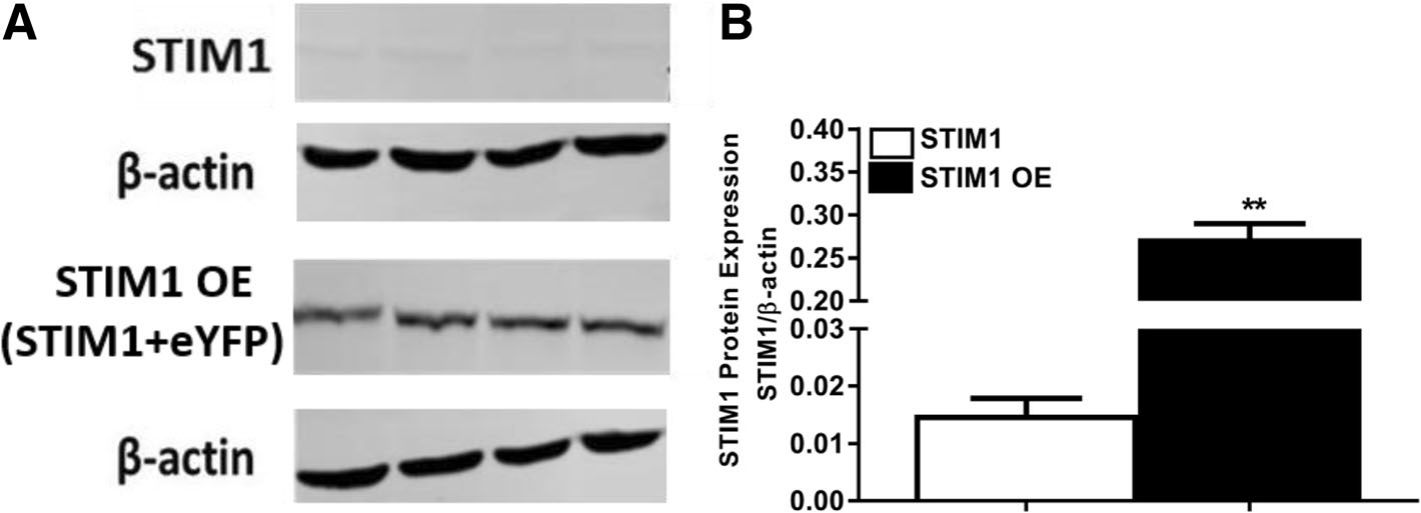
STIM1 protein expression in STIM1-OE EpCAM(+)CD133(+) Huh-7 cells. Shown are (**a**) STIM1 control (77 kDa) vs. STIM1-OE bands (STIM1 OE; STIM1 + eYFP ~ 103 kDa) and (**b**) cumulative data of STIM1 protein expression levels. STIM1 band intensities were normalized to β-actin’s (STIM1/β-actin; **p < 0.01, Student *t*-test, unpaired data, *n* = 4)

In STIM1-OE EpCAM(+)CD133(+) Huh-7 cells, STIM1 protein level was significantly higher (3 fold) than that of the control (**p < 0.01, Student *t*-test, unpaired data, Fig. 4a and b).

**Fig. 5:**
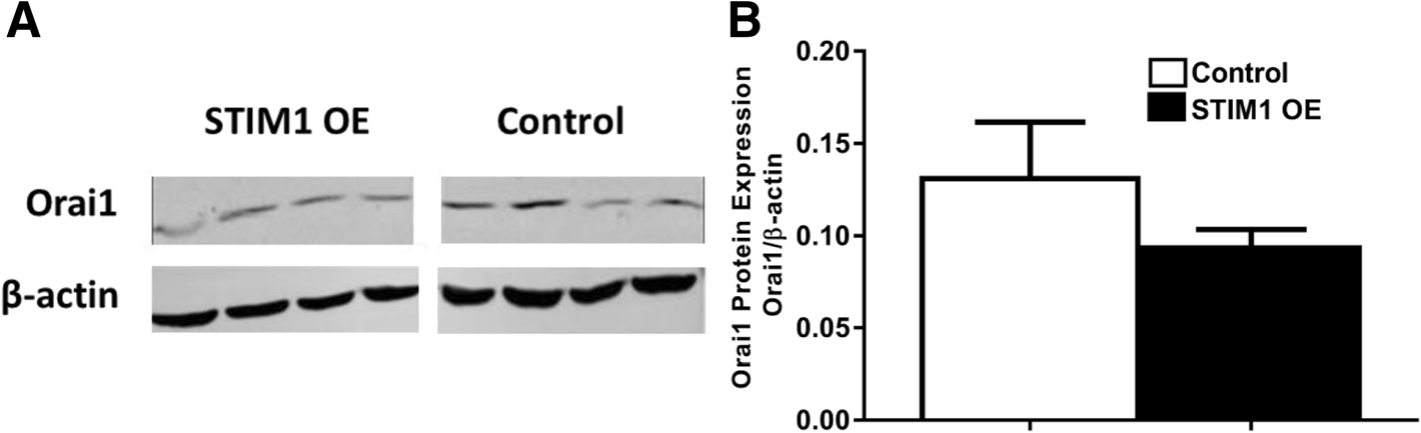
Orai1 protein expression in STIM1-OE EpCAM(+)CD133(+) cells. Shown are (**a**) Orai1 (33 kDa) bands in WB analysis and (**b**) cumulative data of Orai1 protein expression levels. Orai1 band intensities were determined according to Orai1/β-actin ratios. (N.S., Student *t*-test, unpaired data, *n* = 4)

Although not statistically significant, the Orai1 protein level was lower in STIM1-OE samples comparable to that of the control (Fig. 5).

**Fig. 6:**
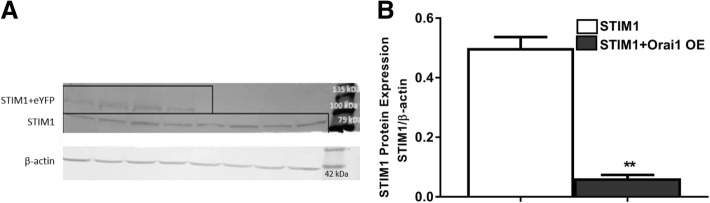
STIM1 protein expression in STIM1 + Orai1-OE EpCAM(+)CD133(+) cells. Shown are (**a**) STIM1-OE (STIM1 + eYFP ≈103 kDa) vs. STIM1 (77 kDa) bands in WB analysis and (**b**) cumulative data of STIM1 protein expression levels. STIM1 band intensities were normalized to β-actin’s (STIM1/β-actin; **p < 0.01, Student *t*-test, unpaired data, n = 4)

STIM1 protein levels decreased in STIM1 + Orai1-OE EpCAM(+)CD133(+) cells (*p* < 0.01, Fig. 6) possibly due to administration of STIM1 and Orai1 plasmids together.

### Intracellular Ca^2+^

**Fig. 7:**
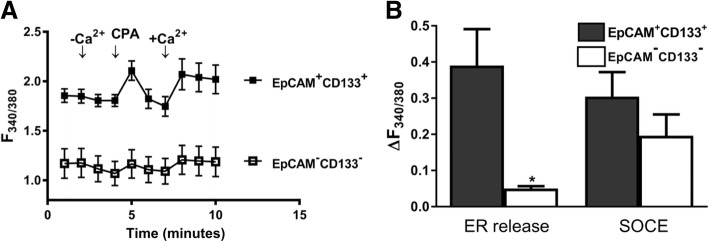
Changes in ER Ca^2+^ release and SOCE in EpCAM(+)CD133(+) and EpCAM(−) CD133(−) cells. Shown are (**a**) EpCAM(+)CD133(+) vs. EpCAM(−)CD133(−) (99%, Fig. 1) cells (Mean ± S.E.M.) and (**b**) cumulative data of ER Ca^2+^ release and SOCE (*p < 0.05, Student *t*-test, unpaired data, n = 4–6). ΔF_340/380_: changes in intracellular Ca^+ 2^_i_ levels

Intracellular basal Ca^2+^ levels were significantly higher in EpCAM(+)CD133(+) comparable to those of EpCAM(−)CD133(−) cells. Although Ca^2+^ elevation due to ER release was significantly higher in EpCAM(+)CD133(+) cells (*p < 0.05, Student *t*-test, unpaired data, *n* = 4–6), SOCE was not altered (Fig. 7).

**Fig. 8:**
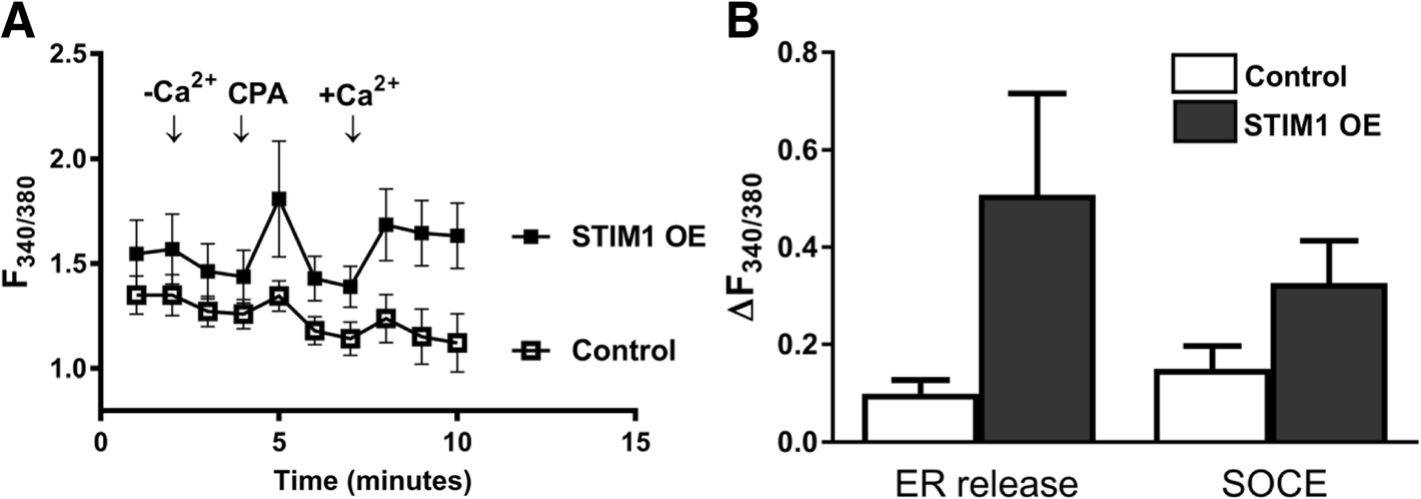
Changes in ER Ca^2+^ release and SOCE in STIM1-OE EpCAM(+)CD133(+) cells. Shown are (**a**) control vs. STIM1-OE EpCAM(+)CD133(+) (64%, Fig.1) cells (Mean ± SEM) and (**b**) cumulative data of ER Ca^2+^ release and SOCE (Student *t*-test, unpaired data, n = 4–6). ΔF_340/380_: changes in intracellular Ca^+ 2^ levels

**Fig. 9:**
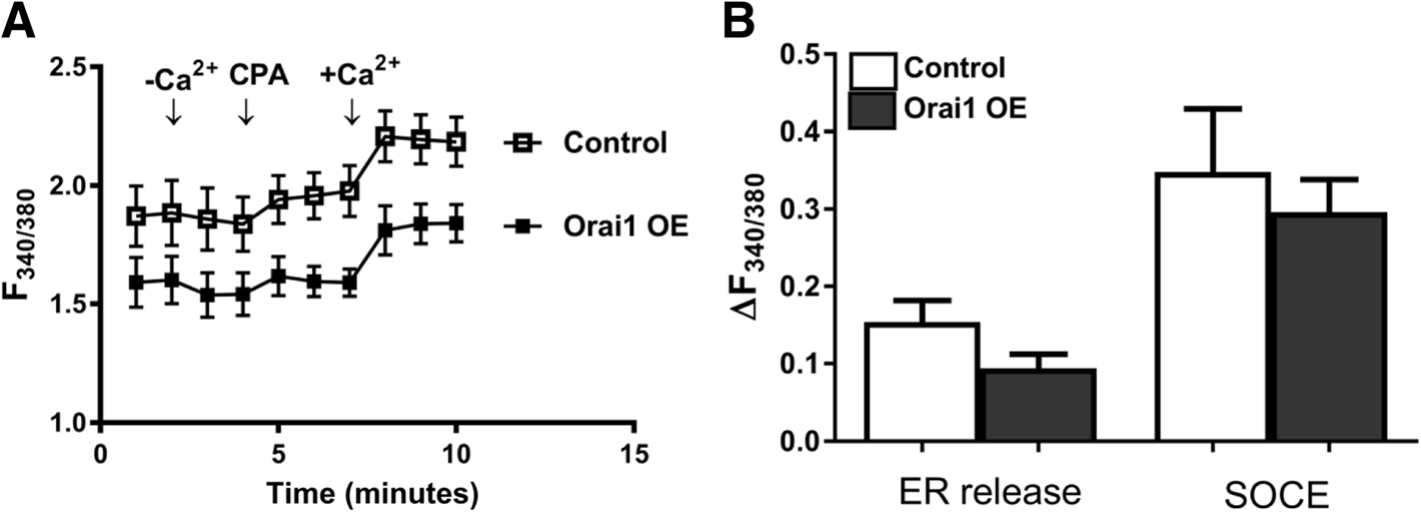
Changes in ER Ca^2+^ release and SOCE in Orai1-OE EpCAM(+)CD133(+) cells. Shown are data from (**a**) control vs. Orai1 OE EpCAM(+)CD133(+) (64%, Fig. 1) cells (Mean ± S.E.M.) and (**b**) cumulative data of ER Ca^2+^ release vs. SOCE in (Student *t*-test, unpaired data, *n* = 5). ΔF_340/380_: changes in intracellular Ca^+ 2^ levels

**Fig. 10:**
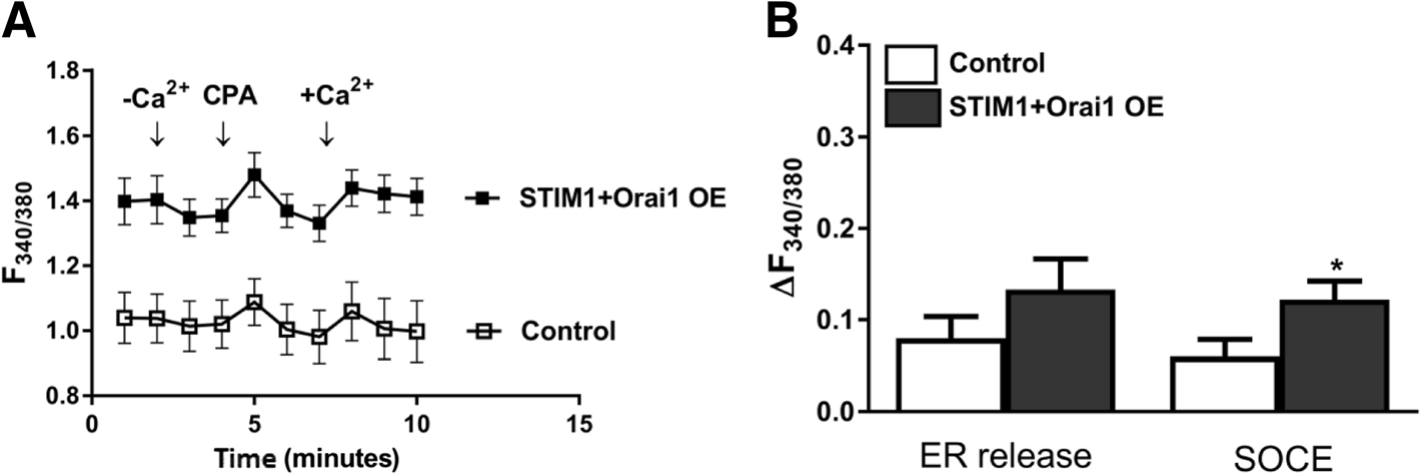
Changes in ER Ca^2+^ release and SOCE in STIM1 + Orai1 overexpressed EpCAM(+)CD133(+) cells. Shown are (**a**) control vs. STIM1 + Orai1-OE EpCAM(+)CD133(+) (64%, Fig. 1) (Mean ± S.E.M.) and (**b**) cumulative data of ER Ca^2+^ release and SOCE (*p < 0.05, Student *t*-test, unpaired data, n = 5). ΔF_340/380_: changes in intracellular Ca^+ 2^ levels

Although there was an apparent increase both in ER Ca^2+^ release and SOCE, the data did not reach statistical significance in STIM1-OE EpCAM(+)CD133(+) cells (Fig. 8). No significant change was observed in basal, ER Ca^2+^ release (expected) and SOCE, possibly due to increased coupling efficiency between depleted ER and Orai1. Although ER Ca^2+^ release and SOCE decreased in Orai1-OE EpCAM(+)CD133(+) cells, the data were not statistically significant (Fig. 9). SOCE increased significantly (*p* < 0.05, Fig. 10) in STIM1+ Orai1-OE cells without any change in ER Ca^2+^ release.

### Cell proliferation patterns in EpCAM(+)CD133(+) and EpCAM(−)CD133(−), STIM1-OE and STIM1 + Orai1-OE EpCAM(+)CD133(+) cells

**Fig. 11:**
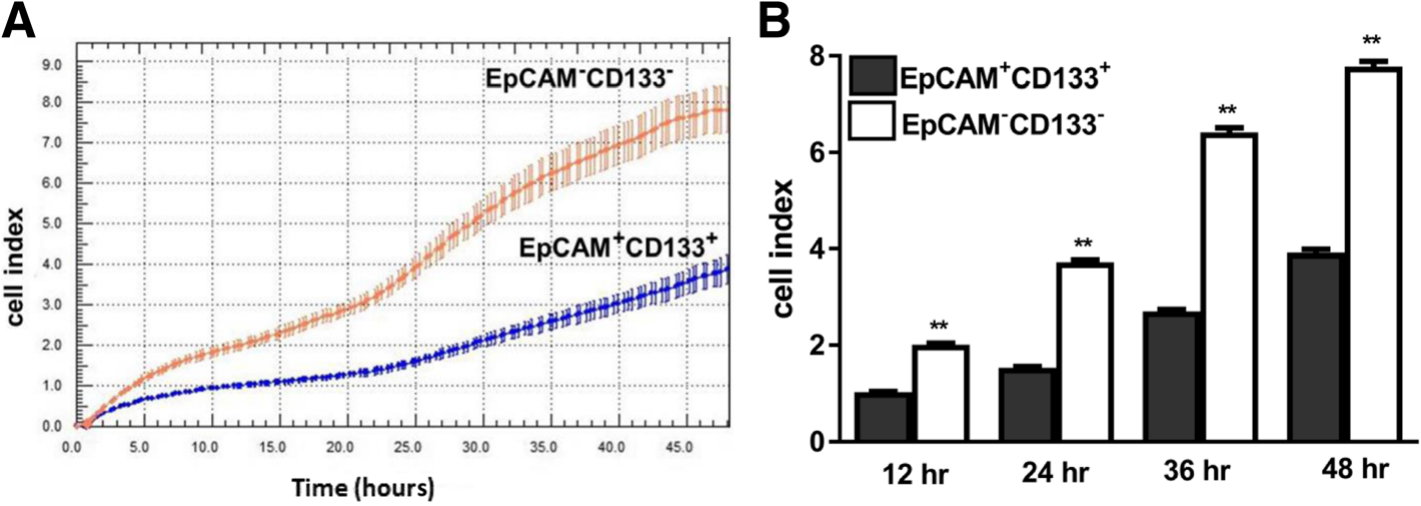
Real-time proliferation patterns of EpCAM(+)CD133(+) vs. EpCAM(−)CD133(−) cells during 48 h. Seeding density was 5000 cells/well. Shown are (**a**) real-time proliferation pattern of EpCAM(+)CD133(+) vs. EpCAM(−)CD133(−) and (**b**) cumulative cell index data (**p < 0.01, Student *t*-test, unpaired data, *n* = 24)

Elevations in impedance (cell index) in RTCA show an increased cellular proliferation rate in real-time. In this study, differences in the cell proliferation pattern were monitored in two groups [EpCAM(+)CD133(+) vs. EpCAM(−)CD133(−) cells and STIM1-OE and STIM1 + Orai1-OE EpCAM(+)CD133(+) cells]. The proliferation rate at 48th h was significantly higher in EpCAM(−)CD133(−) cells comparable to that of EpCAM(+)CD133(+) (**p < 0.01, Fig. 11).

**Fig. 12:**
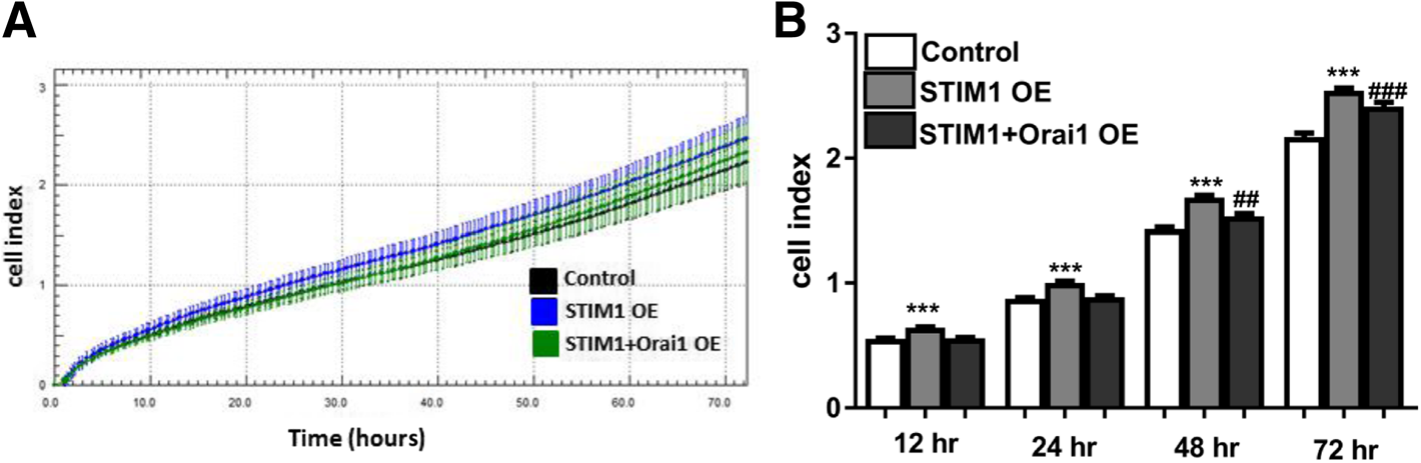
Real-time proliferation patterns of control, STIM1-OE and STIM1 + Orai1-OE EpCAM(+)CD133(+) Huh-7 cells. Cell seeding density was, 5000 cells/well. Shown are (**a**) real-time proliferation pattern of STIM1-OE vs. STIM1 + Orai1-OE EpCAM(+)CD133(+) during 72 h and (**b**) cumulative cell index data (***p < 0.001, ^###^*p* < 0.001, ^***##***^*p < 0.05*; *control vs. STIM1-OE, ^**#**^control vs. STIM1 + Orai1-OE, Student *t*-test, unpaired data, *n* = 32)

We also monitored the effects of STIM1 and STIM1 + Orai1 overexpression on cell proliferation in EpCAM(+)CD133(+) Huh-7 cells. Comparable to the control, STIM1-OE cells at 72nd h showed the highest proliferation rate (*p* < 0.01, Fig. 12); higher than that of STIM1 + Orai1-OE.

**Fig. 13:**
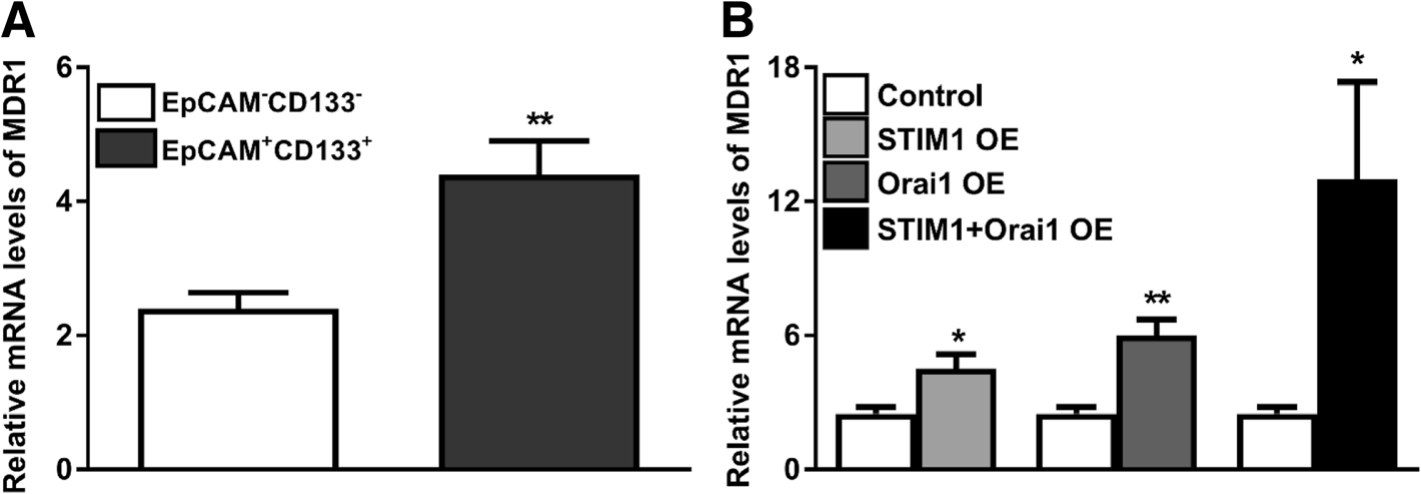
MDR1 mRNA expression levels. Shown are (**a**) EpCAM(+)CD133(+) vs. EpCAM(−)CD133(−) cells, (**b**) control vs. STIM1, Orai1 and STIM1 + Orai1-OE EpCAM(+)CD133(+) cells (Target gene/18S rRNA x10^2^; **p < 0.01 and * *p* < 0.05, Student t-test, unpaired data, *n* = 4)

The difference in multidrug resistance gene (MDR1) expression between tumor-initiating cells and tumor cell lines as well as the effects of STIM1 and Orai1 overexpression on MDR1 transcription in a number of experimental settings were investigated as increases in SOCE appeared to be associated with chemoresistance [44]. MDR1 mRNA levels were significantly higher in EpCAM(+)CD133(+) cells that in EpCAM(−)CD133(−) cells (**p < 0.01, Student *t*-test, unpaired data, *n* = 4, Fig. 13). Elevation of MDR1 in EpCAM(+)CD133(+) was potentiated by inducing STIM1 or an Orai1 expression and drastically increased (6-fold) by STIM1 + Orai1 overexpression (*p < 0.05, Student *t*-test, unpaired data, n = 4, data not shown).

## Discussion

In addition to being involved in intracellular Ca^2+^ homeostasis mechanism of non-excitable cells, SOCE appears to be operational in hepatocellular carcinogenesis [26]. In this study, the role of SOCE components, STIM1 and Orai1, reportedly involved in intracellular Ca^2+^ regulation was investigated on Huh-7 TICs expressing cell surface antigens EpCAM and CD133 through monitoring intracellular Ca^2+^ dynamics (ER Ca^2+^ release and SOCE), proliferation and MDR1 expression responsible partly for drug resistance. High intracellular Ca^2+^ concentration comprises toxic and proapoptotic conditions for cells. Excessive Ca^2+^ is buffered by certain proteins (e.g., calsequestrin and calreticulin) inside ER and by mitochondria. ER Ca^2+^ release and SOCE are significantly higher in EpCAM(+)CD133(+) cells comparable to that of EpCAM(−) CD133(−).

Overexpression of STIM1 and Orai1 is shown in many cancer types like prostate cancer, breast cancer, glioblastoma and hepatocellular carcinoma [33]. More specifically, STIM1 overexpression is commonly seen in HCC [26, 39]. Among the three overexpression groups of EpCAM(+)CD133(+) Huh-7 cell subpopulation in our study, STIM1-OE showed the highest ER Ca^2+^ release. As STIM1 has Ca^2+^ binding EF hand domains located on the intracellular part of ER [45], its overexpression may buffer more Ca^2+^, leading to more Ca^2+^ available to be released from ER following SERCA blockade by CPA. STIM1 is the key initiating molecule in SOCE. After ER depletion, as a sensor of ER Ca^2+^ content, STIM1 accumulated in ER membrane closely located to PM with Orai1. At this ER and PM junctions, STIM1 interacts with Orai1 as a result SOCE is activated [46]. Lower ER release and SOCE in Orai1 OE EpCAM(+)CD133(+) Huh-7 cells comparable to the control cells could be due to changes in coupling stoichiometry between STIM1-Orai1 for SOCE [47]. Higher levels of the PM channel subunit (Orai1) might decrease effective coupling of two molecules (STIM1 and Orai1) yielding SOCE inhibition. Increases of ER release and SOCE in STIM1 + Orai1-OE EpCAM(+)CD133(+) cells, show presence of appropriate coupling stoichiometry between STIM1 and Orai1 for SOCE as both molecules are freely available for random interaction [32]. Similar SOCE elevations were also seen in STIM1 + Orai1-OE and only STIM1-OE DU145 (prostate cancer cell line) and HEK (human embryonic kidney) cells, respectively [40, 48, 49]. Overexpression of Orai1 in DU145 and HEK cells also inhibited SOCE, as observed in EpCAM(+)CD133(+) cells in our study [40, 47, 48].

TICs tended to remain in a quiescence state [50]. These EpCAM(+)CD133(+) cells have slow proliferation rates comparable to that of EpCAM(−)CD133(−) [49, 51] as was also observed in the present study. This may support their survival strategy in a cytotoxic environment [34, 52, 53]. The higher proliferation rate of STIM1-OE cells, comparable to that of STIM1 + Orai1-OE cells, showed that upregulation of these two genes (STIM1 and Orai1) suppresses the cell division/proliferation possibly through attenuated Ca^2+^ buffer capacity of ER. Again, the significantly higher proliferation rate observed with STIM1-OE cells over that of EpCAM(+)CD133(+) cells overexpressing both STIM1 and Orai1 (present data) confirms the poor prognosis of several cancer types with overexpressed STIM1 [54–56].

Cancer cells show resistance to chemotherapeutic treatments. This may result from drug inactivation, changing drug targets, DNA damage repair, and efflux of drug from cells by ABC transporters [57]. Because of the upregulated ABC transporters, cancer cells can pump chemotherapeutics out of the cell [58]. The “slow and steady” feature might also be maintained by higher MDR1 (an ABC transporter family member) expression. Upregulated MDR1 in EpCAM(+)CD133(+) Huh-7 cells in the present study is also in accordance with the increased MDR1 gene expression in lung cancer [59], ovary cancer [60], osteosarcoma [61] and glioblastoma’s [62] cancer stem cells. The signaling pathways (JAK/STAT, PI3K/AKT, MAPK/ERK), which take place in drug resistance, are regulated by Ca^2+^/calmodulin dependent protein kinase II (CaMKII), suggesting an interaction between Ca^2+^ and MDR mechanisms in liver cancer [38].

## Conclusions

Based on the higher proliferation rates observed in STIM1-overexpressing EpCAM(+)CD133(+) Huh7 cells compared to that of STIM1 + Orai1-OE constructs, one may conclude that HCC stem cells might undergo a phenotypical switch process from a quiescent to proliferative stage by increasing ER Ca^2+^ buffering capacity due to higher levels of Ca^2+^-binding protein, STIM1. Furthermore, one may also speculate that increased ER Ca^2+^ buffering prevents Ca^2+^- dependent processes in mitochondria localized within the ER microenvironment by inhibiting Ca^2+^ uptake via low affinity/high capacity Ca^2+^ uniporter of mitochondria.

## Data Availability

The datasets used and/or analyzed in the present study are available from the corresponding author.

## Abbreviations

ABC: ATP-Binding Cassette
BSA: Bovine Serum Albumin
CD133: Clustering Domain 133 (Prominin 1)
CRAC: Calcium release-activated calcium
DMEM: Dulbecco’s modified eagle medium
EpCAM: Epithelial cell adhesion molecule
ER: Endoplasmic reticulum
FACS: Fluorescence-activated cell sorting
FITC: Fluorescein isothiocyanate
HBS: HEPES-buffered saline
HCC: Hepatocellular carcinoma
MDR: Multidrug resistance
OE: Overexpressed
PE: Phytoerythrin
PM: Plasma membrane
RTCA: Real-time cell analyzer
SDS-PAGE: Sodium dodecyl sulfate polyacrylamide gel electrophoresis
SOCE: Store-operated calcium entry
STIM: Stromal interaction molecule
TIC: Tumor-initiating cells
